# The evolution of the concepts of ‘primate culture’ in Western science

**DOI:** 10.1007/s10329-026-01244-5

**Published:** 2026-02-13

**Authors:** Malene Friis Hansen, Agustin Fuentes

**Affiliations:** 1 Aarhus Institute of Advanced Studies, Aarhus University, Aarhus, Denmark; 2 Department of Anthropology, Princeton University, Princeton, USA

**Keywords:** Concepts, Dynamic assemblages, Japanese primatology, Natural sciences, Primate culture, Social sciences, Western primatology

## Abstract

While most scholars across the social and biological sciences acknowledge that human culture is distinctive in the comparative context there is widespread acknowledgment that some form of culture does occur in other animals, including in many species of primates. Here we outline key historical patterns and the changes in primatology and the behavioural sciences regarding the concepts of primate culture. In the contemporary moment new methods and approaches are emerging as the field of inquiry matures. Our assessment is that continued and expanded inclusion of methods and theory from both the social and the natural sciences is beneficial, and that a further development of a form of primate ethnology for the comparison and analysis of primate cultures is needed. With a better understanding of the complexities of the primate cultural dynamic, scientists may better be able to model their evolutionary dynamics and consider aspects of primate cultures in conservation action.

## The concepts of primate culture

While a broad range of scholars acknowledge that human culture is certainly distinctive in the comparative context (Fuentes 2018; Read 2011; Tomasello 2014) there is widespread acknowledgment that some form of culture does occur in other animals, and certainly in many species of primates (Whiten 2000; Whiten et al. 2021). In this review we do not engage in the larger academic debate of whether animals, including non-human primates (primates), have culture or not. Rather, our goal is to provide a historical framing and a timeline of changing views and assessments of research on primate culture, including terminology and a discussion of the changes, and what this has meant, and does mean, for the study of primate culture as a scholarly endeavour in the 21st century.

The concept of ‘primate culture’ has been a source of academic debate for nearly a century. However, the effective international academic emergence of the study of primates and culture was launched with the first English publications on primate “sub/pre” culture by the Japanese scholars Imanishi and Kawamura in 1957 and 1959. The debates around primate culture continue across multiple disciplines today but have altered and expanded according to the changes in knowledge bases and research targets, moving from investigations of “do primates have culture,” to research on different transmission mechanisms of primate cultural elements and eventually to dynamics of the construction of primate cultures. Current work on primate culture shares the common goal of increasing understanding of behaviour that can be considered cultural and in-depth analyses of cultural dynamics in the primates. Here we offer a brief summary of how the theory and method around the study of primate culture emerged and what the current moment offers.

## The initial framing: Kinji Imanishi and the Japanese approach to “karachua”

As early as 1887, a publication on stone tool-use in long-tailed macaques (*Macaca fascicularis)* appeared in the western scientific literature (Carpenter 1887) but it was not described explicitly as a cultural behaviour. Since then, there have been numerous academic accounts of primate behaviour that match aspects of the various definitions of culture used for humans. However, for much of the 20th century culture was seen as one of the elements that distinguish humans from other primates and other animals. This blanket assertion made it difficult for early researchers and other scholars to describe behaviours they observed in other primates as “primate culture.” Also, because the preponderance of definitions of culture was specifically geared towards the details of human culture (especially language ability), it proved difficult to employ any of them directly in the study of anything that one might term as “primate culture” (i.e. Imanishi 1952 in DeTroy et al. 2024; Kummer 1971).

To overcome this obstruction the Japanese anthropologist/ecologist/primatologist Kinji Imanishi (1952) (in DeTroy et al. 2024) provided two different words for culture, “bunka” and “karachua”. Bunka is the Japanese word for culture and is used to refer to all the cultural manifestations of humans, such as language and playing music. Karachua is the Japanese phonogram of the English word culture and presents a broader conceptualization of culture. Karachua is defined as learned acquired behaviour that can only exist and be transmitted in groups that have continuous group-living (Imanishi 1952 in DeTroy et al. 2024) and is therefore distinguished from innate inherited behaviour. Imanishi (1952) writes (in DeTroy et al. 2024; p.366): “*It is clear that we must recognize culture (karachua)*,* which we once considered as human lifestyle*,* in animals closely related to us*,* like monkeys and other mammals*,* at least to some extent*.” Unfortunately, Imanishi’s critical contribution regarding animal culture was not translated from Japanese to English until 2024 (DeTroy et al. 2024) but was acknowledged, and used, by Japanese researchers for over 70 years prior to that. While such work did sporadically enter into the English-language primatology from the 1950s on, Imanishi’s view remained as a minority perspective or unexamined/unnoticed by a majority of the non-Japanese primatological community until the 21st century. Imanishi defined primate culture as a learned behaviour that is socially transmitted, an inclusive and non-reductionistic definition that only began to emerge as a consensus across the Western primatological literature in the early 2000s.

Imanishi was a part of the Primate Research Group in Japan and conducted research on the Japanese macaque (*Macaca fuscata*). His colleagues included the primatologists Kawamura, Itani, and Kawai among others. Their assessments and insights on primate “karachua” came from research into the Japanese macaques, for example, the sweet-potato and grain washing macaques on Koshima island (Imanishi 1957; Kawai 1965) and the candy-eating macaques in Takasakiyama (Itani 1958). The Japanese researchers were able to observe the beginning of these idiosyncratic social behaviours and their subsequent transmission through the macaque groups naming them *subhuman or prehuman culture*,* or subculture* (in English translations). With the publication of the findings of the Japanese researchers in English, some Western scientists became aware of their discoveries. Writing in American Anthropologist in 1959 (p.595), J.E. Frisch called these reports of karachua “protoculture,” stating, “*To the extent to which culture is equated with learned*,* traditional behavior*,* monkeys appear to have indeed much more “culture” than anthropologists have often thought*. With the “acculturation of the propagation of subculture” as primatologist Kawamura (1959) explained it, observed in several groups, Kawamura was able to show differences in propagation of “subculture” between the different groups. In 1960 (p.402), Imanishi wrote “*Accordingly if an experimental oikia is created successfully from individuals of two different oikiae*,* it may in course of time reveal whether and how much behavioral characteristics derive from heredity and/or culture.*” (Oikia is here the primate social group). Kawamura (1959; p.48) attempted to describe the exact mechanisms of social learning in the propagation of primate cultural behaviours as passive acquisition via social facilitation as opposed to active teaching “*….in general the learning is wholly one-sided on the part of the receiver*,* and the transmitter takes no active part in it*.”

In 1965, another Japanese scholar, the zoologist Masao Kawai, provided data on the vertical (from parent to offspring) and horizontal (between members of same generation) transmission of the behaviour in the same group concluding that it indeed was cultural, naming it a *pre-culture*. Kawai (1965; p.24) discussed the social learning mechanisms stating that “*Macaques are generally believed inferior in the ability of imitation* (Yatabe 1945), *but judging from the propagation of these two behaviors*, *it may be acceptable that in a natural troop imitation through daily lives is made rather intensively*.” This may be the first time imitation is mentioned for wild primate populations in the primatological literature. Kawai also suggested that matched dependent behaviours might have been in play here, with the frequency of the behaviour increasing according to the frequency of rewards.

The Japanese scholars used the prefixes (in translation here) pre/sub/proto for culture and subhuman primates for primates. The psychologist/primatologist C. R. Carpenter argued against the use of the word subhuman and recommended using non-human instead in his comments to a publication from 1960 by Imanishi (Carpenter, 1960 in Imanishi, 1960). Imanishi (1960; p.406) responded by saying “*I am interested in the origin of culture*,* and*,* in my usage*,* the “subhuman” level of primates is nearly equivalent to the “subcultural” level of primates*.” As Carpenter also comments, information might be lost or misunderstood when translating scholarly work to English from other languages and from other scientific traditions than the Western one (Carpenter, 1960 in Imanishi 1960).

The Japanese researchers’ work, especially in the 1950–1970 s, pioneered critical methods and theory in the field of primatology in regard to primate culture (Asquith 1989; Frisch 1959; Imanishi, 1960). They were the first to provide a definition of primate culture, the first to provide evidence of cultural transmission, and the first to discuss social learning mechanisms in cultural transmission. They were also the first to employ focal animal observations, a key method that enabled these crucial findings.

### Western approaches to primate culture

C.R. Carpenter, a pioneer in naturalistic field observations of primates, commented that “*Clearly*,* there is a need to progress from qualitative descriptions*,* though without neglecting the refinement of them*,* to the formulation of quantitative expressions which accurately represent behavior*,* social interactions*,* characteristics of group organizations*,* population dynamics*,* and ecological factors. Field studies of primates*,* including man*,* will eventually require appropriate field theory and field mathematics*” (Carpenter, 1960 in Imanishi 1960; p.402), Imanishi and colleagues did in fact provide much of the mode of systematic methods called for by Carpenter. However, the semi-quantitative and systematic approach to animal behaviour sampling used today was not initially formalized until 1974 by behavioural ecologist Jeanne Altmann, setting the stage for further quantification of primate behaviour (Altmann, 1974). It is in the context of Carpenter and his early field observations, and of the work of Altmann, that the western study of primate behaviour, and its relations to primate culture, is framed.

In 1928 (p.341), the anthropologist Alfred L. Kroeber hinted at the possibilities of primate culture: “*The study of the anthropoids*,* however*,* yields grateful and valuable corroboration. Cultureless these higher primates are; but with reactions and faculties closely akin to our own*,* and manifesting at least some measure of the basal psychic ingredients which enter into culture.”* Kroeber and Kluckhohn (1952) reviewed 164 definitions of culture presenting a single multifaceted definition as a synthesis. They included “human” as a key signifier in the synthesis definition but offered no possibility for the inclusion of other animals. Years later, in 1964, the first widely accepted evidence for, and possibility of, culture in free-ranging primates was explicitly discussed in the Western academic literature. Primatologist Jane Goodall (1964; p.1266) presented several observations of tool use in chimpanzees (*Pan troglodytes*) and concluded that “*It therefore seems probable that the use of sticks*,* stems and leaves for the specific purposes described here represents a series of primitive cultural traditions passed on from one generation to the next in the Gombe Stream area*.” In this publication she noted that the observations were conducted at an “artificial feeding site” similar to the methodologies incorporating provisioning used by the Japanese researchers a decade earlier. Kummer (1971), using core conceptual frameworks from Biology, defined non-human animal (animal) culture as “*a behavioral modification induced by the social environment*” and further explained that in groups with the same habitat and gene pool, any behavioural differences not genetically determined/facilitated are likely cultural. If only habitats differ, behavioural differences are due to ecological adaptive modifications. If gene pool differs, the behavioural differences are due to phylogenetic modifications. The transmission mechanisms and possible categories of cultural behaviours in animals were, according to Kummer, unknown and lacked meaningful criteria for distinction (Kummer 1971; p. 13). Kummer’s discussion of non-human culture was based on his training as an ethologist and extensive field research on Hamadryas baboons (*Papio hamadryas*) and via reviewing research from other species, such as the Japanese macaques. Culture in captive primates was also discussed in this time, with comparative psychologist E. W. Menzel and colleagues (1972; p.168) concluding that “*In summary*,* the present authors believe that culture-like phenomena are much more widespread than has been previously thought*” after conducting captive studies on chimpanzees showing transmission of socially learned behaviours and asserting that the chimpanzees displayed cultural or protocultural behaviour.

The physical anthropologist Mann (1972) published a core discussion on the differences between humans and primates related to culture. Mann noted that the anthropologist Goodenough (1971; p.19) stated that “*Culture can be seen as standards of appropriate behaviour*,* learned by the individual*,* which allow him successfully to interact within his own social and group milieu. It is apparent that this definition also applies to the social system of the non-human higher primates*.” Relying on Goodenough’s definitions of cultural artefacts as the learned material manifestations, Mann stated “*Thus*,* employing this approach to culture*,* the conclusion is reached that there is little difference between hominid and non-hominid higher primates*.” Mann continued “Therefore, *we may no longer speak of ‘culture’ as being exclusively hominid. Yet*,* it is valid to distinguish a behavioural complex dependent on tools*,* which is a hominid domain*. *I submit the term ‘human culture’ for this system*,* and suggest that it be defined as a set of learned behaviours that modify the environment and are crucial to the survival of the species. This distinction is a foundation on which to reconstruct hominid and human cultural origins*” (1972:p.382). Mann presents a distinction common today, that of some boundary between human and primate culture. For Mann human culture was crucial for human survival (a sort of human cultural niche), and primate culture not necessarily so.

The appeal to distinguish between human and primate culture (or animal culture) is pervasive in the primate culture literature. In a review of the nervous system and cultural capacities of humans, psychiatrist Fabrega (1977; p.423) compared findings to primates and said “*Nonetheless the existence of such cognitive behaviors -* concepts of self and spatial memory organization *- among the higher primates*,* the obvious preadaptations for a form of language and communication*,* and indeed the stylistic attributes of their group behavior is what has led to the claim of their possessing “culture”* and then referred to Kummer (above) and Parker (1976). Fabrega discussed the body/brain relations of humans and primates and their connections to cognitive abilities, which became a reoccurring theme in relation to culture within primate culture researchers, especially among comparative psychologists.

Behavioural ecologists William McGrew and Caroline Tutin (1978) provided an operational definition, laying the foundation for cultural research in primates for the remainder of the 20th century. They offered eight assessments/characteristics that were quantifiable and could be applied to traits or behaviours that might be cultural. These were innovation, dissemination, standardization, durability, diffusion, tradition, non-subsistence and natural adaptiveness. It is noteworthy that the eight principles did not include the perspectives and framings of the Japanese scholars, although their work is referred to. McGrew and Tutin were concerned that any measure or study of cultural behaviour should not occur in provisioned primate populations, because provisioning was deemed artificial and thus believed to interfere with “natural” processes in primate populations. Also, the human influence on the non-provisioned primate populations under observation for culture was not allowed to exceed the level of “human forager groups,” for example, and primate study populations should not inhabit agricultural lands as that too was seen as too much human-based impact on the “natural” state of the primates’ lives. This operational and reductionistic (and hierarchal) definition rendered the critical work by Japanese researchers, such as the sweet potato washing of Japanese macaques, not truly “cultural,” because of the human influence. In conjunction with the operational approach of McGrew and Tutin, the concept of “Method of Exclusion” for investigation of non-human cultures solidified the reductionistic approach. In such a perspective, a behaviour can only be assigned as cultural if genetic and environmental variables can be excluded as causal factors (Galef 1976; Nishida 1987; see also Kummer 1971).

This framing, the eight characteristics and the method of exclusion, was critical in setting the western research agenda for primate culture for decades. Around this time (the 1970s/early 1980s), the split between scholars who focused on non-human cultures (animal behaviourists and cognitive psychologists) and those who focused on human culture (largely anthropologists and other social scientists) grew and the cross-fertilization of ideas between them reduced until the start of the 21st century. For example, in response to McGrew and Tutin, the physical anthropologist Sherwood Washburn and the cultural anthropologist Burton Benedict (1979) argued against the use of “culture” as a term or frame in reference for primates, at least in the sense of equivalency to the process/dynamic that is human culture. Harkening back to Kroeber and Kluckhohn, Washburn and Benedict emphasized that linguistic processes, language itself, is a central feature in all definitions, and processes, of human culture and as non-humans lacked language it was not accurate to assume that the common term “culture” was applicable to both human and non-human dynamics.

In 1979, McGrew and colleagues published the first cross-population comparison of a primate culture, calling it an ethnology of the chimpanzee. A few years later, McGrew also found cross-cultural variation in diets between different chimpanzee populations (McGrew 1983), and in 1992, McGrew published a book on chimpanzee culture focusing on material culture further documenting tool use in this primate. He claimed at the time that chimpanzees were the only consistent tool users and provided evidence of tool use by chimpanzees in their free-ranging populations. In 2002, this was corroborated by anthropologists and primatologists Bernardo Urbani and Paul Garber, who concluded, according to their knowledge at the time, that monkeys (and not the great apes) only use tools in captivity.

Between the late 1970s and the early 2000s, primate culture as a topic of study, and its mechanisms, was the source of much debate and research focus in primatology and in animal behaviour. Within this debate, the social learning abilities of primates, especially imitation, became a main focus. The focus on transmission of behaviours and the capacity, or lack thereof, for imitation became the dominant field of study, with the documentation of actual behaviours that could be termed cultural and the possibility of structured cultures, and what such structural dynamics might mean for primate societies taking a back seat. In the 1980s–90s, animal behaviourists and philosophers, largely trained as psychologists, such as Whiten, Tomasello, Byrne, and others, took the lead in the focus on primate culture, investigating and discussing the social mechanisms behind cultural transmission. In this psychologically oriented frame, the assumptions that culture is best transmitted through imitation, with emulation and social facilitation playing only minor roles, became prominent. Much research then turned to focus on whether or not primates were truly capable of imitation, with initial evidence of imitation in the study of human-reared chimpanzees (*Pan troglodytes*) (Tomasello et al. 1993a) and the possibility of imitation in wild gorillas (Byrne and Byrne 1993). Psychologist Whiten (2000) provided a full review of the research on imitation and an overview of several methods of social learning for the transmission of culture referring to Whiten and Ham (1992). Psychologists Cecilia M. Heyes and Bennet G. Galef Jr. (1996) also provided a comprehensive compilation concerning the mechanisms of social learning and their implications gathering the insights of most experts working on the topic at that time. In parallel, primatologist Michael Huffman (who trained with Japanese scholars) and colleagues continued the holistic Japanese approach in collaboration with Japanese and other scholars focusing on long-term research on the cultural phenomena of stone handling in Japanese macaques (Huffman et al. 2010).

In 1996, the cognitive anthropologist and philosopher Dan Sperber argued for a naturalistic approach to social science, recommending taking an interdisciplinary approach to cultural research. He argued that defining and understanding culture writ-large (in humans and in other animals) was too often limited to an approach that emphasized a purely functional Darwinian approach assuming cultural units as analogous to genes (e.g. Dawkins 1976; 1982; Cavalli-Sforza and Feldman 1981) but not including innovations from anthropology and cognitive psychology. Sperber emphasized the propagation and representation of culture as a core feature, calling it “the epidemiological approach”.

By 1998 (p.322) McGrew summarized the “state of the art” in “cultural primatology” asserting that “*In conclusion*,* primate culture in the broad sense seems likely to be a stem trait dating back at least to the last common anthropoid ancestor. As such*,* it predates all the derived traits of hominization*,* that is*,* bipedalism*,* enlarged and lateralized cerebral cortex*,* lithic technology*,* symbol use*,* and so on. To paraphrase Gertrude Stein culture is*,* culture is culture*,* in a variety of species*,* if culture is taken to be group-specific behavior that is at least partly acquired from social influences. At the same time*,* human culture does not equal chimpanzee culture does not equal Japanese monkey culture. Each species is unique*,* but as more and more data accumulate*,* cross-species differences look more to be ones of degree*, *not kind*.” McGrew also introduced the term” Cultural Primatologist” and it became a sub-division in primatology with the growing acknowledgement of primate culture as a central aspect of primate studies. A keystone review by Whiten and the majority of primary investigators in chimpanzee research projects was published in 1999 displaying the diversity and extent of chimpanzee cultural behaviours across groups, populations and subspecies. This review solidified the chimpanzee as a prime target species for primate cultural behaviour investigation and inspired many to conduct research into other primate species and investigate if they hold the same diversity of cultural behaviours as the chimpanzees (see e.g. Sinha 2005 for bonnet macaques (*Macaca radiata*), Malaivijitnond et al. 2007 for long-tailed macaques and Ottoni and Izar 2008 for capuchins (*Cebus spp*.).

While multiple ways to define culture continued in use, and the McGrew and Tutin framework remained powerful. Evolutionary biologist Kevin Lala(nd) and zoologist Will Hoppit (2003:p.151) reiterated and streamlined what has now become the standard core definition of primate culture: “*group-typical behavior patterns shared by members of a community that rely on socially learned and transmitted information*”. This dominant definition is often combined with the more operational definition from psychologist Dorothy Fragaszy and anthropologist Susan Perry (2003) that requires cultural behaviours to be group and/or population specific, prevalent in the group and/or population across age and sex classes, persistent over generations, and socially transmitted and maintained. In 2003, Fragaszy also published “Making Space for Traditions” defining traditions as prevalent, persistent and socially transmitted, yet not group or population specific differentiating it in one area from cultural behaviours (Fragaszy, 2003).

## Primate “culture” in the 2000s

By the early 2000s many primatologists and a range of animal behaviour researchers were regularly using the term “culture” as per the Lala(nd) and Hoppit definition in their work and including it as an aspect of their investigations, see for example the overview of primatologist Sinha (2005) for bonnet macaques. In 2004, biologist Hal Whitehead and colleagues proposed the inclusion of cultural behaviours when defining populations for conservation action, arguing for a revision of the definition of the “Evolutionary Significant Unit” (ESU). The ESU label is given to a population of organisms considered sufficiently distinct as to be considered its own unit for conservation purposes. Contemporary conservation action is often based on ESUs as opposed to species as an ESU is not always equivalent to the entirety of a species’ range, but can be also a subspecies, variety, or geographic population. Whitehead et al. (2004) argued for cultural ESUs, discussing the differences in stable vertically transmitted cultures vs. transient/adjustable horizontally transmitted cultures and their significance for the ability to change and evolve in populations and thereby adapt or not in rapidly changing environments. They stated that “*For a range of non-human animals*,* culture is a vital determinant of phenotype*,* and so how the animals interact with humans and our cultural artifacts. Thus*,* culture should be an integral element of the conservation biology of these species*.” They further state that *“non-human culture is not just ‘‘chimpanzees /dolphins/elephants reading poetry’’*,* it is the source of survival skills fundamental to the daily lives of these animals*.” (Whitehead et al. 2004; p.434). Whitehead and colleagues denote an expansion of the view of animal culture from a view of culture as the emergence of novel social traditions and “non-genetically linked” behaviours to acknowledging culture as a core aspect of the specific group/population’s survival-critical, or adaptive, evolved, behaviour repertoire. Two years later, environmental scientist Ryan (2006; p.1323) offered an operational framework for incorporating and considering culturally significant units in conservation actions, noting that “*Culturally significant units (CSUs) present the conundrum of opposing strategies: maximizing variation versus preserving uniqueness*” and discussed how, for example, translocations and reintroductions fail to consider culture in the populations affected.

The view of specific animal cultures as adaptive units emerged in concert with another line of animal culture research: the ”Disturbance Hypothesis” (van Schaik 2002). This approach viewed human incursion into other animals´ habitats and lives, via habitat loss and hunting pressure, for example, as having negative effects on the innovation and transmission of social traditions. Thereby reducing the behaviour repertoire, potentially harming the evolved or “natural” relationship between a group of animals and their local environment. Evolutionary anthropologist Carel P. Van Schaik employed the term tradition instead of culture, referring to the framing of ‘tradition’ proposed by Fragaszy (2003). Both the cultural ESU/CSU and the “disturbance hypothesis” views of primate culture focused on local varieties of cultural behaviour as potentially adaptive results of animal-environment histories and interactions and thus critical factors in the survival of those groups. This urges conservation practitioners to carefully consider culture in the animals they are assessing. For example, zoologist Hjalmar S. Kühl and colleagues (2019), presented arguments for chimpanzee conservation, using the Disturbance Hypotheses and the effects on chimpanzee culture, arguing for Culturally Significant Units as key targets of conservation in chimpanzee populations. This view remains a common one today amongst animal culture researchers.

In 2010, Whitehead expanded the discussion regarding conserving animal cultures and the animal populations that create cultures, arguing that animals with increased innovative skills, forms of neophilia, and complex social learning in anthropogenic environments, such as synanthropes (species that are able to utilize human resources and share habitats with humans), could potentially have an increase in the diversity in behaviour that could become cultural in the zone of interface with humans. This moved the debate from a sole focus on the preservation of extant systems (the disturbance hypothesis) to the possibility that animals have cultural agency, and that behavioural flexibility may modify and/or innovate cultural processes to engage with changing environments. More recently, biologists Nicole Creanza, Oren Kolodny and Marcus W. Feldman (2017) also argued that the rapid shifts in anthropogenic landscapes might be facilitating cultural transmission in other animals.

## Cultural transmission/construction

To date, the modes of transmission and the structural dynamisms of cultural processes remain a focal point for scientific investigation aside from the conservation focus. In particular, the debate on whether primates (and other animals) have cumulative culture is central. Cumulative culture is when a culture increases in efficiency and/or complexity over time via the process of passing down beneficial knowledge and skills across generations, which leads to more complex cultural innovative and cumulative traditions via the “ratcheting effect” (Mesoudi and Thornton 2018; see also Boyd and Richerson 1996 and Tomasello 1999). Up until recently cumulative culture was also thought to only occur in humans (Tomasello 1999; Lala(nd) 2017). However, multiple scholars over the past decade or so have argued that cumulative culture occurs in primates as well. For example, behavioural ecologist Schofield et al. (2018) reviewed 50 years of research in the sweet-potato washing behaviour of Japanese macaques and concluded that they have cumulative culture, “at least in a simple form”. Interestingly, anthropologist Jessica Rosien and colleagues report a similar innovation of food washing in a population of long-tailed macaques and further argue that “cultural” opting out of food-washing could be adaptive for higher-ranking individuals (Rosien et al. 2025). Primatologist and ethologist Christophe Boesch also discussed the probability of chimpanzee cumulative culture in 2012 (Boesch 2012; p.56–66) and so did ethologist JB Leca and colleagues at the same time for the Japanese macaque (Leca et al., 2012).

The debate around the capacities and likelihood of cumulative culture and the link to collective knowledge was recently summarized by Whiten and colleagues (2022:p.2). They conclude that: “*Collective knowledge may change over time when the same group members solve the same task repeatedly*,* or across partial turnovers in group membership*,* amounting to the emergence of a group culture. This may in turn extend to cultural transmission of collective knowledge across generations. Any additional extensions in such collective cultural knowledge may in turn evidence cumulative culture”*.

Cultural transmission and cultural evolution have also received much focus in captive primate research, for example, on Guinea baboons (*Papio papio*) in laboratories. Here baboons through a pattern reproduction task created distinct cultural behaviours with systematic structure, lineage specificity and performance increase over time (Claidiére et al. 2014).

The transmission debate, beyond the cumulative culture aspect, often revolves around acknowledging the difficulties with determining which behaviours are cultural and how they are acquired. Evolutionary biologist Caroline Schuppli and van Schaik (2019) recommended focusing on assessing if behaviours are learned and transmitted socially and moving away from the Method of Exclusion. For example, they showed that almost all immature orangutan (*Pongo* sp.) behaviours are socially learned and transmitted. Recent research into capuchin (*Sapajus libidinosus)* tool use over time, showed different stages of tool use in correlation to environmental changes (Falótico et al., 2019). Work by the anthropologist Charmalie Nahallage and Huffman (2007) with Japanese macaques demonstrated that merely being exposed to a stone handling model(s) was a key social stimulus influencing the acquisition of such behaviour. This research supports including multiple factors, including environmental and demographic factors, in analysis of primate culture, moving away from the notion of ‘culture” being those social traditions or behaviours not directly genetically or environmentally mediated.

A summary of current debates and practices in primate cultural study was offered by the anthropologist Langlitz (2018; p.17), who writes:” *Part of the ensuing and still ongoing scientific controversy surrounding non-human primate cultures has revolved around the question of how broadly or narrowly to define the relevant forms of social learning. For example*,* can we speak of the cultural transmission of behavior if an individual figures out how to use a tool left behind by another – or does it require the exact imitation of a carefully observed series of actions? Whatever psychological mechanism enables animals to learn from each other*,* the result is that different groups will do things differently*.” Building on this, Langlitz commented on the disturbance hypothesis suggesting that “*cultural primatology* [ala McGrew] *amounted to an ethnographic salvage operation in the face of anthropogenic mass extinction*.”

The dynamic nature of culture, as highlighted by Langlitz is often overlooked by those scholars looking at primate and other animal cultures. Animal culture is often depicted as static or an endpoint in the ongoing adaptive story, as opposed to an ongoing, unending process by which innovation and change, loss, disruption, accumulation, and individual agency are all simultaneously possible. Today, despite the massive increase in both data and theoretical diversity around the topic, the focus for much of the study of primate culture remains on specific behaviours/traits in local cultures/groups and the debates around active species-specific social learning.

Recently the term “co-culture” was presented by ethologists and primatologists Cédric Sueur and Huffman (2024). Sueur and Huffman argue that cultures can be co-created by different sympatric species through their interactions. They advocate for the inclusion of co-culture in cultural research and conservation actions, especially in urban planning with the myriad of interspecific interactions occurring involving sympatric species, also many primates and humans. We (Hansen and Fuentes 2025) discuss this dynamic and present the term co-construction of behaviour, suggesting that co-constructed behaviours could be a first step before co-cultures. We offer the possibility of co-construction of behaviour between animals and humans as a locus of study. Our examples focus on macaques, a genus that is particularly adept in utilizing human resources and innovating novel behaviours according to environmental changes and human behaviour. Many macaque groups exemplify what Whitehead discussed in 2010 (see earlier) and appear to co-construct cultural behaviours with humans.

The frame of “co-cultures” (Sueur and Huffman 2024; Sueur et al. 2025) and the context of contemporary research on primate culture, invites researchers to expand conceptualizations, and observations, of how primates interact with their environments and sympatric species, challenging the primate research community to expand modalities of researching primates and take more seriously a comprehensive and dynamic view of primate cultures.

## The impact of culture concepts and the dynamic assemblages of primate culture research

Over a decade ago primatologist Tatyana Humle and behavioural ecologist Nicholas Newton-Fisher (2013) discussed primate culture as more nuanced and offered a specific definition from McGrew (2003), “*culture is the way we do things*” and a way to shape other theoretical framings. There is no debate that everyday life and routine is seen as a part of human culture (Brown 2004; Kroeber and Kluckhohn 1952). Could primate culture also include, and be seen in, the daily life and routines of primate populations? The quotidian experience differs between groups and populations of the same species, influenced by ecological circumstances. But what if the histories of specific groups, the personalities and patterns of agency within the populations, largely experienced and transmitted socially, are also central facets of what we could term a given primate culture? This supposition aligns well with the definition of culture of Schuppli and van Schaik (2019), and resonates with the group differences observed by Kawamura in 1959 and the discussions and proposals of Hansen and Fuentes (2025), Sueur and Huffman (2024), van Dooren et al. (2025), Langlitz (2018) and McGrew (1998). Such a conceptualization of primate culture suggests expanding beyond relying on specific “items” or “units” of behaviour as the main constituents of culture to include both active and passive transmission and acquisition of a more diverse array of behaviours facilitated, structured, and shaped by the lived experiences of the individual primates of the group and the history of the group itself. Across changing environments, even at the micro-scale, and with different individuals, personalities, and relationships in primate groups, cultures as assemblages may change dynamically over time. Expanding our view of primate culture to include recent advances advocates for an interdisciplinary approach to cultural primatology.

Most cultural primatologists implement very strict operational definitions for primate culture derived from McGrew and Tutin (1978) and Fragaszy and Perry (2003). When working under such strict disciplinary paradigms, we may miss crucial aspects of primate culture, as Schuppli and van Schaik (2019) and environmental philosopher and anthropologist, Thom van Dooren and colleagues (2025) note. Reductionism and functionality are key aspects of the Natural Sciences and should not be ignored, however combining the systematic quantitative approach of the Natural Sciences with diverse and often richly qualitative work frames in the Social Sciences (and even the Humanities) could provide a more holistic toolkit for investigating primate (and other animal) culture. While it may be sometimes difficult to draw directly from ethnographic methods in Cultural Anthropology and to engage in an interdisciplinary way with all areas of inquiry, having input from Cultural Anthropologists and having them engage with primate culture research offers both an expansion of possible toolkits and also supports primatologists from the natural sciences deploying the comparative approach in assessing and defining what constitutes primate culture (see for example Dore et al. 2017, Malone 2022; Riley 2019). Such an integration could remove some of the obstacles while still providing scientifically rigorous results. The field of Ethnoprimatology (Dore at al. 2017; Fuentes 2012; Malone 2022; Riley 2019) offers some examples of this approach and animal behaviour scholars are engaging these dynamics more frequently. Such scholars include zoologist Claire N. Spottiswoode and anthropologist Brian M. Wood (2023) and their research in interspecific communication and collaboration between honeyguides and humans, and zoologist Cantor et al. (2023) and their research on dolphins and fishermen collaboratory relations in Brazil. This interdisciplinary approach could facilitate thinking of primate culture as more than specific behaviours and the use of material items and expand to include the assessment of the dynamic assemblages (Fuentes et al., in press) that constitute the daily lives and the cultures of primates that fluctuate according to environmental changes, group composition, individual agency, interspecific interactions with sympatric species and a plethora of other processes. A unifying lens across the natural and social sciences is needed, with research into the overlaps and distinctions between primate and human culture using diverse, interdisciplinary, and integrative approaches.

In this article we outline key historical patterns and the changes to the concepts of primate culture, where definitions and methodological approaches have changed substantially over time, but where certain threads remain throughout. Perhaps primate culture is as much the immersive experience of being a part of a group as it is a specific tool use behaviour. In the current moment new methods and approaches are emerging as the field of inquiry matures (Creanza et al. 2017; Sueur and Huffman 2024; Whiten et al. 2022). Moving forward these should include the interplay between different cultural behaviours, between the behaviours and the environment, between the individuals and their groups and the environment, and here especially reconsidering what defines a group or a population. In environments where different sympatric primate species (including humans) coexist, a group or a population of interest could also be formed by different species. Primate culture is complex and while the field of inquiry has expanded over the past century, there is still much left to investigate and understand. Our assessment is that continued and expanded inclusion of methods and theory from both the social and the natural sciences (as discussed above) is beneficial. This could, for example, involve greater incorporation of ethology to measure the behaviours and individuals/groups in question, a substantial ecological analyses to look at the interplays with the environment, modified ethnographic methods to qualitatively observe the lives of the groups and thereby the cultures that they live in, and finally, further development of a form of primate ethnology for the comparison and analysis of primate cultures (Dore et al. 2017; Malone 2022; Setchell et al. 2017). With a better understanding of the complexities of the primate cultural dynamic, primate scientists may better be able to consider them in conservation action (Brakes et al. 2025a, b; van Dooren et al. 2025).

**Table 1:** Key concepts within the field of cultural primatology with definitions and references

Concept	Definition	Reference
Co-culture and co-construction of culture	Co-construction of culture by sympatric species	Sueur and Huffman 2024
Collective knowledge	“*Collective knowledge refers to the ways in which knowledge is distributed and shared among members of* *the organization*”	Lam 2000 (p. 491); Whiten et al. 2022
Cultural learning	Social learning at different levels	See e.g. Tomasello et al. 1993b
Cumulative culture	Culture increases in efficiency and/or complexity over time	See e.g. Boesch 2012; Schofield et al. 2018
Emulation	Learning from the results of the behaviour of another individual	See e.g. Whiten 2000
Facilitation	Behaviour improvement through the presence of others	See e.g. Whiten 2000
Horizontal transmission	Transmission between members of same generation	See e.g. Cavalli-Sforza 1986
Imitation	Copying the behaviour of another individual	See e.g. Whiten 2000
Second inheritance system	Cultural inheritance	Whiten 2017
Tradition	“*A tradition is a behavioral practice that is shared among members of a group; is performed repeatedly over a period of time (that is*,* it is enduring); and depends to a measurable degree on social contributions to individual learning for its appearance in new practitioners*”	Fragaszy 2003 (p. 61)
Vertical transmission	Transmission from parent to offspring	See e.g. Huffman 1984;1996 and Cavalli-Sforza 1986

Caveat: The review presented here focuses primarily on accounts from published peer-reviewed scientific journals and academic books, largely in the English language or in English translation, and is therefore deprived of accounts and concepts of primate culture conveyed through other forms of knowledge sharing and in other languages. This initial review is an assessment of primarily European and North American science, plus key Japanese science,and therefore the range of knowledges from other cultural systems and ways of knowing about primate culture is not included. Engagement with and inclusion of such other sources/knowledges would greatly diversify and enhance our understanding of the topic and is thus a goal of future work.
